# Supplementary material for: Promoting healthy lifestyles among nurse trainees: Perceptions on enablers and barriers to dietary and physical activity behaviours
Source: PLoS One. 2022 Jun 24;17(6):e0270353. doi: 10.1371/journal.pone.0270353 (PMC9231720; doi:10.1371/journal.pone.0270353)
Supplement: S1 File — (DOCX) [file pone.0270353.s001.docx]

**Table 3. Motivating factors for healthy diet and physical activity behaviours.**

| Sub-themes | Additional responses obtained on the different themes |
| --- | --- |
| Individual (Intrapersonal) Factors | |
| Self-discipline | Eating time  *“That one I will advice myself. I will make sure I wake up and eat at the right time. When in the morning I have to take this and when in the afternoon I have to take this and the evening so I will advice myself”* (IDI, female, underweight BMI) |
|  | *“In this school the time a proportionate to a lecture is very, very poor. They can schedule a time like from 7.30am and me too I wake up like 6.30am … So, I think what I have to do is I have to be waking up early to prepare something to eat”* (IDI, Male, normal BMI) |
| Dietary knowledge | *“vegetables it all depends on what it is going to do and I will take it”* (IDI, Female, normal BMI) |
|  | *“maybe getting to know recipes of foods that do without oil”* (IDI, Female, normal BMI) |
| Physical environment | |
| Geographical access/availability | *“I will say the convenience the location where I found myself and most at times banana is kind of seen there all the time so I just find myself buying banana just to take some fruits but in the case I go to the market and I see other fruits I can afford I just buy them and I take it”* (IDI, Female, normal BMI) |
|  | *“oh it depends I already told you in Ho the standard of living is very high so that one also depends on income”* (IDI, Male, normal BMI) |
|  | *“Okay, I think it all has to do with availability of the foods. If we can let’s say have variety of foods especially the vegetables and then the fruits. In almost vantage points. Everybody can access. I mean it will go along to affect our dietary lifestyles. Meaning I will be eating much more healthily.”* (IDI, Male, underweight BMI) |
| Social environment | |
| Social support | *“Okay, maybe if someone encourages me to like do exercises like that maybe it can help me engage in more activity.” (IDI, Male, underweight BMI)* |
|  | *if there are games on campus with friends involved I will. University of Health and Allied Sciences (UHAS) is full of stress and you don’t have much time to play or exercise. Else you put your grade point average (GPA) at risk” (IDI, male, normal BMI)* |

**Table 4. Barriers to healthy diet and physical activity behaviours.**

| Sub-themes | Additional responses obtained on the different themes |
| --- | --- |
| Physical Environment | |
| Accessibility | “*on campus is different I don’t normally buy fruits, but I buy fruits when I come across them, but I don’t buy fruits that much because you are short of money and you want to be careful with your spending, so you don’t call home frequently*” (IDI, Male, Normal BMI) |
|  | “*In Ghana right now, it is very hard for you to get the vegetables and those fruits they are very expensive aahaa. So, with me with my income (feeding money) I don’t think I can afford that*” (IDI, Male, normal BMI) |
| University characteristics | |
| Studies/ academic activities | *“…academic activities too can prevent me because if I am having lectures I can also not have time for that. At times Saturdays that we are supposed to go for jogging and other things lectures will be scheduled for that time and during exams time we don’t have time to go and engage in such activities…”* (IDI, Male, Normal BMI) |
|  | *“is time, sometimes we don’t have lecture here but other campus, so if you want to do some exercise. You need to wake up around 4:00am”* (IDI, Male, Normal BMI) |
| Student associations | *“Yeah if there is no like keep fit on campus for that one it will prevent me from doing it and even the house as I was going for it someone motivated me. Every morning the fellow comes to my house to come and call me. Because I know that this lady will be coming every day I make up my things ready so when she comes I don’t waste her time I just go out and we all go together. but if she is not coming I also feel relaxed but if it is on campus and the keep fit clubs are always available then there is motivation, we know the importance of it I think it will always keep me going.”* (IDI, Female, Normal BMI) |
| Social Environment |  |
| Upbringing | *“When I don’t have anything doing I stay indoors so that is the only thing that will prevent me from walking. But apart from that when I have lectures I walk and when I want to buy something from the town I walk to the market to buy it. But when I am at home and don’t feel like doing anything that means I am indoor.”* (IDI, Male, Overweight BMI) |
