# Supplementary material for: Promoting healthy lifestyles among nurse trainees: Perceptions on enablers and barriers to dietary and physical activity behaviours
Source: PLoS One. 2022 Jun 24;17(6):e0270353. doi: 10.1371/journal.pone.0270353 (PMC9231720; doi:10.1371/journal.pone.0270353)
Supplement: S2 File — (DOCX) [file pone.0270353.s002.docx]

**Lifestyle Behaviours and its Implications on Body Mass Index**

**Screening Questionnaire**

**Date of interview ---------------/--------------/------------**

**Introduction**

Hello. My name is __________________________. Thank you for sparing time for this interview. We are conducting this study to examine the relationship between lifestyle behaviours and its implications on body mass index (BMI). This study seeks to gather data from university students on perceived motivators and barriers in engaging in healthy lifestyle behaviour and also understand differences if any with regards to lifestyle behaviours by BMI categories. Prior to the in-depth interviews (IDIs) this questionnaire will be administered to enable us collect your background information and anthropometric measures. The anthropometric measures will aid us to appropriately determine your body mass index for inclusion into the study.

1. Age in completed years……………….
2. Sex …………………………………….
3. Are you in a campus/ hostel residence? Y/N
4. Marital status……………………………
5. Employment status………………………
6. Religion………………………………….
7. Ethnicity………………………………….
8. Body weight (kg)………………………...
9. Height (cm)………………………………
10. BMI (kg/m^2^) ……………………………...

Thank you for your time.
