# Supplementary material for: Promoting healthy lifestyles among nurse trainees: Perceptions on enablers and barriers to dietary and physical activity behaviours
Source: PLoS One. 2022 Jun 24;17(6):e0270353. doi: 10.1371/journal.pone.0270353 (PMC9231720; doi:10.1371/journal.pone.0270353)
Supplement: S3 File — (DOCX) [file pone.0270353.s003.docx]

**Lifestyle Behaviours and its Implications on Body Mass Index**

**In-depth Interview Guide**

**Date of interview ---------------/--------------/------------**

**Introduction**

Hello. My name is __________________________. Thank you for sparing time for this interview. We are coming from the health research institute university of Health and Allied Sciences and we are conducting this study to examine the relationship between lifestyle behaviours and its implications on body mass index (BMI). This study seeks to gather data from university students on perceived motivators and barriers in engaging in healthy lifestyle behaviour and also understand differences if any with regards to lifestyle behaviours by BMI categories. It is hoped that the information will also be important in improving healthy lifestyle behaviours among the youths and reduce obesity a risk factor to non-communicable diseases (NCDs). It is also imperative that you give honest responses. I will be guiding the conversation and my colleague here will be writing down what is said but as it may not be possible to write everything down on paper, my colleague would like to tape this discussion. Let me stop for a moment to respond to any question you may have [**Please** **Pause – Answer Any Questions].**

May I turn on the tape recorder? [**Turn On Tape Recorder)**

**Opening questions**

1. Describe activities you have engaged in within the last 24hrs
2. Are the mentioned activities usual or one off?

(Probe for reasons why and if they are usual only at school or weekends and home)

**Perceptions on BMI**

1. What do you think is an ideal body size for people your age?

(Probe for reasons)

1. What do you think about your own body size?

(Probe for reasons why she/he thinks it is ideal or not)

**Self-assessment of lifestyle behaviours**

1. What do you think about your
2. eating habits (fruit, vegetables, oil, salt, eating time etc)
3. physical activity
4. water consumption
5. hours of sleep
6. alcohol intake
7. smoking

**Perceived facilitators to healthy lifestyles**

1. Can you describe factors that will enable you to [identifying facilitators]:
2. Eat healthily (fruits and vegetables, less oil, less salt, avoid late eating)
3. Engage in regular physical activity
4. Drink water regularly (7 glasses or more)
5. Sleep adequately (7-8 hours)
6. Smoke
7. Consume alcohol

**Perceived barriers to healthy lifestyle behaviours**

1. Could you describe why you might not be able to [identifying obstacles]:
2. Eat healthily (fruits, vegetables etc)
3. Engage in regular physical activity
4. Drink water regularly
5. Sleep adequately
6. Smoking
7. Consume alcohol

**Conclusion**

1. Will you change any lifestyle if you would have to do it over again?

(Probe for which lifestyles and for reasons)

Would you like to ask any question about this study?

Thank you for your time.
